# MiRNA-24 downregulates *KLF6* affecting STAT3 protein expression and phosphorylation regulating melanogenesis in cashmere goat coat

**DOI:** 10.5713/ab.24.0824

**Published:** 2025-06-10

**Authors:** Baoyu Zhang, Runlai Liu, Yuxin Zhao, Xinyu Li, Huixian Su, Shufang Li, Jianping Li, Huaizhi Jiang, Qiaoling Zhang

**Affiliations:** 1 College of Veterinary Medicine, Jilin University, Changchun, China; 2 College of Animal Science and Technology, Jilin Agricultural Science and Technology University, Jilin, China; 3 College of Animal Science and Technology, Jilin Agricultural University, Changchun, China

**Keywords:** Cashmere Goat, *KLF6*, Melanogenesis, MiRNA-24, STAT3

## Abstract

**Objective:**

This study revealed that miRNA-24 downregulates *KLF6*, regulating melanogenesis in the coat of Cashmere goats.

**Methods:**

The correlation between miRNA-24 and coat color was determined by quantitative reverse transcription polymerase chain reaction (RT-qPCR). GO/KEGG analysis and bioinformatics tools were used to screen for target genes. Targeted interactions were confirmed using the dual luciferase assay. The expressions of miRNA-24 and *KLF6* on different colored skins of Cashmere goats, and the *KLF6* expression in B16-F10 cells transfected with miRNA-24 mimics/inhibitors/*NC* were detected by RT-qPCR, Western Blot, and Immunofluorescence. To investigate the pathway that *KLF6* influenced, *KLF6* was silenced by RNA interference (RNAi), and the melanogenesis pathway’s gene expression was examined. The expression and phosphorylation of STAT3 protein in the pathway were inhibited. RNAi and Stattic were utilized to downregulate its protein expression and phosphorylation for detecting tyrosine expression. BALB/c mice were subcutaneously injected with antagomiRNA-24, a miRNA-24 inhibitor. The influence of miRNA-24 on the downregulation of *KLF6* in relation to melanogenesis was examined.

**Results:**

MiRNA-24 is related to coat color. *KLF6* was chosen as the target gene, and the targeted interaction was validated. The expression trends of miRNA-24 and *KLF6* in the skin inversely correlated. The mRNA and protein expression of *KLF6* were altered in B16-F10 cells after transfected. These suggested that miRNA-24 downregulates *KLF6*. Silencing of *KLF6* resulted in the downregulation of genes linked to melanogenesis, including *WNT1*, *WNT2*, *PRKACA*, *MAPK1*, *TYR*, and *STAT3*. The suppression of protein and phosphorylation levels of STAT3 decreaseed the mRNA level of *TYR*, suggesting that *KLF6* influences *TYR* expression by modulation of STAT3 expression and phosphorylation. BALB/c mice injected with antagomiRNA-24 showed increased melanin content and decreased *KLF6* expression.

**Conclusion:**

MiRNA-24 regulates melanogenesis in the Cashmere goat coat by downregulation of *KLF6*, thus mediating STAT3 expression and phosphorylation.

## INTRODUCTION

The cashmere came from the outer layer of Cashmere goats, with an average fiber diameter of 15–16 microns. Indicators like cashmere’s length and diameter are valued in the market, but there is also a growing demand for kinds colors for cashmere. The production and distribution of pigments by melanocytes influenced the cashmere color development. The base layer contains melanocyte stem cells, which divide and grow into mature melanocytes [1]. Tyrosine and other raw materials are used to synthesize eumelanin and pheomelanin et al. [2]. Then they are combined in various ratios and delivered to epidermal keratinocytes via cell synapses to produce skin pigmentation [3]. To achieve pigmentation in cashmere animals, dendrites frequently deliver melanin to the inner and outer root sheath cells of hair follicles [4]. Therefore, the formation of cashmere color depends on the melanogenesis and pigmentation process. The activity and concentration of melanin synthase and substrates are two factors that affect the melanogenesis process. UVA increases intracellular calcium ion mobilization to change signal transduction in melanocytes, affecting the activity of key enzymes involved in melanogenesis [5]. MFSD12, an input protein for melanin synthesis, influences the creation of eumelanin [6]. Furthermore, several signaling mechanisms control melanin synthase activity. Classic regulatory pathways for melanogenesis include the Raf/MEK/ERK, the Wnt/β-Catenin, and the α-MSH/cAMP pathway. In addition, non-classical pathways, such as the protein kinase C dependent pathway, ultimately affect tyrosinase activity [7].

MiRNA is a non-coding RNA that is essential for regulating the transcription of genes. The miRNA precursor was cleaved by Type III endonuclease Dicer to produce a mature miRNA that is roughly 21 nucleotides long. Mature miRNAs combine to create RNA-induced silencing complexes (RISC) while controlling gene expression [8]. The seed sequence of the miRNA 5’UTR region preferentially detects and attaches to the mRNA 3’UTR after RISC identifies and binds to the target mRNA [9]. This results in regulatory effects by preventing mRNA translation and destruction. It is generally accepted that miRNAs have a role in biological processes like metabolism, apoptosis, differentiation, and cell proliferation. It is also thought that the intricate regulatory network made up of miRNAs and genes influences the processes of pigmentation and melanin synthesis. Manila clamshell color development is influenced by the regulatory network made up of efu-miR-101, mle-bantam-3p, egre-miR-9-5p, sma-miR-75p, and lncRNA [10]. It has been demonstrated that the Dicer-miR92b-ItgaV pathway is the primary signaling pathway for stress-induced hair whitening [11]. By influencing both traditional and non-classical melanogenesis pathways, the regulatory network associated with miRNA influences the activity of elements like tyrosinase. STAT3 can influence *TYR* expression levels through non-classical α-MSH/cAMP/MITF pathways [12], whereas miR-181a-5p and miR-199a in the extracellular vesicles of human amniotic mesenchymal stem cells drastically reduce melanin concentration in B16-F10 cells by blocking *MITF* [13]. Our group’s findings indicated that miRNA-200a promotes the Wnt/β-catenin signaling pathway in melanocytes, suppresses the expression of *WNT5A* and *FZD4*, and eventually enhances melanin synthesis [14]. The research suggests that miRNA plays a significant role in controlling melanogenesis.

Prior research has demonstrated that miRNA-24 regulates several cell growth and development pathways, including Wnt/β-Catenin. By upregulating the expression of the Wnt/β-Catenin pathway gene *CAMK2B* in rat and bovine muscle cells, miR-24-3p has been demonstrated to control the growth of muscle cells in various mammals conservatively [15]. By suppressing Activin, miRNA-24 eventually influences *hALK4* expression and stops hematopoietic stem cells from developing into red blood cells [16]. MiRNA-24 inhibits the growth of hair follicle stem cells by targeting the cell cycle-related enzyme that codes for the gene *PLK-3*. Researchers discovered that it impacts the expression of stem cell regulatory factor *Tcf-3* and interferes with the normal differentiation process of cells [17]. Simultaneously, there was a noticeable loss of hair follicles and hair follicle cysts in the mice created with ectopic expression of miRNA-24 [18]. According to current studies, miRNA-24 also has a role in the MAPK signaling pathway. It prevents P38 phosphorylation and promotes cell proliferation by focusing on and downregulating the expression of genes involved in the MAPK pathway [19]. Additionally, miRNA-24-3p, along with miRNA-146a-5p, miRNA-146b-5p, and miRNA-425-3p, is strongly associated with WNT and MAPK signaling and is substantially elevated in depressive individuals [20]. It has been established that the miRNA-24/VHL/HIF-1α double negative feedback network enhances HIF-1α expression, enhancing the survival and growth of rectal cancer cells [21]. The activating protein STAT3 is less active when the miRNA-24-3p/JAK/STAT pathway is expressed, which ultimately causes T cell death [22].

There is currently a lack of research on how miRNA-24 affects melanocyte growth and development, and nothing is known about how it affects melanogenesis. Investigating how miRNA-24 regulates melanocyte genes and signaling pathways is crucial since melanocytes play a key role in developing animal cashmere color. This study initially confirmed the association between miRNA-24 and the development of cashmere color. To explore its regulatory network, this study screened the *KLF6* gene linked to cashmere color formation. It was discovered that miRNA-24 targets the CUGAGCC sequence in the 3’UTR region of the *KLF6* gene. The inhibitory effect of miRNA-24 on *KLF6* gene expression was then confirmed by analysing the expression of both skin and B16-F10 cells transfected with miRNA-24 mimics/inhibitors/*NC*. After silencing *KLF6* expression, the expression of STAT3 protein involved in melanogenesis was significantly reduced, and its phosphorylation level decreased. When silencing STAT3 protein expression and administering Stattic (phosphorylation inhibitor), the mRNA level of *TYR* decreased, indicating that miRNA-24 downregulates *KLF6*, mediating STAT3 protein and phosphorylation, affecting Melanogenesis. Lastly, antagomiRNA-24 supplementation of BALB/c mice resulted in decreased melanin levels and increased *KLF6* gene expression in the BALB/c mice’s skin. The investigations filled the mechanism of miRNA-24 acting on melanogenesis by confirming that miRNA-24 influences melanogenesis in Cashmere goats coat by downregulating *KLF6*.

## MATERIALS AND METHODS

### Animal sample collection

This study was approved by The Tab of Animal Experimental Ethical Inspection, JLU (KT202402385). Four mature male Cashmere goats with black and white hair were obtained from the breeding center located in Baishan City, Jilin Province. Following collection, the scapular skin was covered in tin foil and preserved at −80°C. For this experiment, 20-day-old female BALB/c mice were bought from Liaoning Changsheng Biotechnology (Shenyang, China). Each of the two groups—the AntagomiRNA-24 group and the control group—had four mice in a row. The 0.5 OD antagomiRNA-24 and phosphate-buffered saline (PBS) were subcutaneously injected into each BALB/c on the back daily for seven days. On the eighth day, take the cashmere from the back of the mouse, cover it with tin foil, and store it at −80°C.

### Quantitative reverse transcription polymerase chain reaction

TRIgent reagent (Mei5bio, Beijing, China) was used to extract the RNA from the animal skin and cells. The miRNA Design V1.01 software was used to generate the quantitative reverse transcription polymerase chain reaction (RT-qPCR) primers for miRNA-24, as shown in Table 1. As indicated in Table 2, the target gene primers were created using the NCBI database and SnapGene software. Jilin Kumei (Changchun, China) synthesized primers. The cDNA of the target gene and miRNA were obtained using the Reverse Transcription Kit (Innovagene, Changsha, China). The experimental system was according to the real-time fluorescence quantitative PCR kit (Innovagene). Pre-denaturation at 94°C for 2 min was the first step. This was followed by the 40-cycle of denaturation at 94°C for 15 s, annealing at 55°C for 40 s, and extension at 72°C for 25 s. Lastly, the system’s dissolution conditions were used to end the experiment.

### Prediction and screening of target genes

The target gene of miRNA-24-3p was obtained through the miRDB (https://mirdb.org/) and Targeted Scan Human 8.0 (https://www.targetscan.org/vert_80/) bioinformatics online websites. Gene IDs can be obtained on the DAVID website (https://david.ncifcrf.gov/home.jsp). The biological functions and involvement pathways associated with targeted genes were examined by KOBAS 3.0 (http://bioinfo.org/kobas/) and Metascape (https://metascape.org/gp/index.html#/main/step1).

### Dual luciferase assay

The vector of psiCHECK-2 used in this experiment was to confirm the targeting effect. The primers of psiCHECK-2-KLF6-WT (wild-type) and psiCHECK-2-KLF6-MUT (mutant-type) had been generated by the SnapGene program and synthesized by Jilin Kumei Biotechnology, the primers sequences were shown in Table 3. The mutant binding site was designed using the point mutation method. The double-stranded linked target gene fragment was annealed by the program of 95°C for 5 min and 37°C for 30 min. Xho I and Not I (Takara, Beijing, China) restriction endonucleases were then employed to cut at 1,643 bp and 1,647 bp in the vector. Using T4 ligase (Takara), the recovered vector fragment and target gene fragment were ligated for an entire night in a metal bath at a constant temperature of 16°C. The vector was transformed into *Escherichia coli* (*E. coli*) DH5α competent cells for positive clone screening and handed over to Biotech (Changchun, China) for sequencing. After the plasmid was extracted using an endotoxin-free plasmid extraction kit (Omega, Norcross, GA, USA), 96-well plates were used to subculture HEK-293T cells with a 70%–90% growth density. The specified transfection steps were carried out per Lipofectamine 2000 (Thermo Fisher Scientific, Wuhan, China) after the cell concentration was more than 70%. The dual luciferase reporter gene detection kit from Promega (Beijing, China) was then used to detect fluorescence values in cells that had been transfected for 24 h.

### Protein extraction and Western blotting

RIPA lysis buffer (Biomed, San Diego, CA, USA), adding phosphatase inhibitor and protease inhibitor (Beyotime, Shanghai, China), was used to lyse animal skin on ice for 30 min. After adding B16-F10 cells to RIPA, they were sonicated for 10 min to achieve full lysis. The supernatant was utilized for sample preparation following the BCA measurement of protein content. The gel should be prepared according to the directions on the Thermo Fisher Scientific 12% polyacrylamide gel kit. Add 15–25 μL of sample to each hole, and then conduct electrophoresis under the following conditions: concentrated gel voltage of 90 V for 30 min, separation gel voltage of 150 V for about 1 h. The protein is transferred onto a PVDF membrane using the wet transfer technique, maintaining a continuous current of 400 mA for 25 min. The PVDF membrane was then sealed for 1.5 h, submerged in a 5% bovine serum albumin (BSA)-TBST solution. KLF6 Monoclonal antibody (1:2,500; Proteintech, Wuhan, China), KLF6 Polyclonal Antibody (1:2,500; Proteintech), STAT3 antibody (1:1,000; Abmart, Berkeley Heights, NJ, USA), STAT3 (phospho Ser727) antibody (1:1,000; Abmart) and β-Actin antibody (1:2,000; ZSGB, Beijing, China) were incubated with the bands for a whole night at 4°C. Then, the bands were washed off thrice by TBST for 10 min each time. After labeling goat anti-mouse IgG and goat anti-rabbit IgG with horseradish enzyme (1:3,000; ZSGB, Beijing, China) for an hour at room temperature, the bands were washed off by TBST again. The protein bands were ultimately observed using an ECL chemiluminescence reagent (Biosharp, Anhui, China).

### Cell culture

Melanocytes were substituted by B16-F10 cells in this experiment. B16-F10, one of the cloned sublines from B16 cells, maintains the capacity to express genes relevant to melanin and to manufacture melanin. Cells were cultured in DMEM high glucose basic media, 10% FBS, and 1% penicillin streptomycin. They would be subcultured when the cell density reached 70%–80%.

### Cell transfection

In a 6-well plate, B16-F10 cells were transferred and cultured for the whole night until the cell density reached 70%–90%. The transfection system I for each well was assembled by adding 7.5 μL of lipofectamine 2000 reagent (Thermo Fisher Scientific) and 100 uL of serum-free medium. System II (100 uL of serum-free media, 75 pmoL miRNA and siRNA) was set up after system I had stood for 5 min. After combining each of them, the system would sit for 20 min. Each well was added 200 μL of the matching mixed transfection system and supplemented to 2 mL with serum-free media. Replace the original medium with 10% serum media and resume culturing after 6 h. After 24 h, acquire samples.

### Immunofluorescence

After 24 h of transfection, the cells were treated as follows: fixed with 4% paraformaldehyde solution for 15 min. Subsequently, cells were subjected to permeabilization treatment using a solution containing 0.3% Triton X-100 for 30 min. Next, the cells were sealed with 3% BSA for 1–2 h to reduce the interference of non-specific binding on the experimental results. After the closure is completed, KLF6 primary antibody (diluted at a ratio of 1:20) is evenly dropped onto a glass slide and incubated overnight in a moist incubator at 4°C to allow the antibody to fully bind to the target antigen. The next day, at 20°C, goat anti-mouse IgG H&L (FITC) secondary antibody was dropped onto the glass slide and incubated for 2 h to achieve specific labeling of the primary antibody. Afterwards, DAPI was used to stain the cell nucleus for 5 min for subsequent localization and counting of cells. After staining, use an anti-fading sealing agent to seal the sample to prevent fluorescence signal quenching and ensure the accuracy of image acquisition. Subsequently, the sample was imaged using a fluorescence microscope to record the expression and localization of KLF6.

### Determination of melanin content

The BALB/c mice skin of each group was frozen continuously in liquid nitrogen and ground into powder. After quantification, they were placed in a 1.5 mL centrifuge tube. Each sample tube was added 1 mL of papain (20 mg/mL) and enzymatically hydrolyzed in a constant temperature metal bath at 55°C for 16 h. The liquid with gauze was filtered to obtain crude melanin at 3,540 ×g for 10 min; then, the melanin was washed three times with petroleum ether, anhydrous ethanol, and distilled water until the pH was neutral. After freeze-drying the final melanin sample, each group was supplied with the same quantity of NaOH (0.1 mol/L), which was then dissolved for 12 h at 100°C. Three parallel wells were inserted into each group in a 96-well plate containing the final melanin sample. The absorbance was measured at 500 nm using an enzyme-linked immunosorbent assay reader. The concentration curve of the melanin standard derived from squid ink was used to quantify the melanin content.

### Statistical analysis

This study’s results were shown as the mean±standard deviation of three different experiments, and SPSS 13.0’s one-way analysis of variance and two-tailed student t-test were used for analysis. GraphPad Prism was used to visualize and evaluate the outcomes. The statistical significance was indicated by three symbols: * p<0.05, ** p<0.01, and *** p<0.001.

## RESULTS

### The coat color correlation analysis and target gene screening of miRNA-24

**Figure 1:**
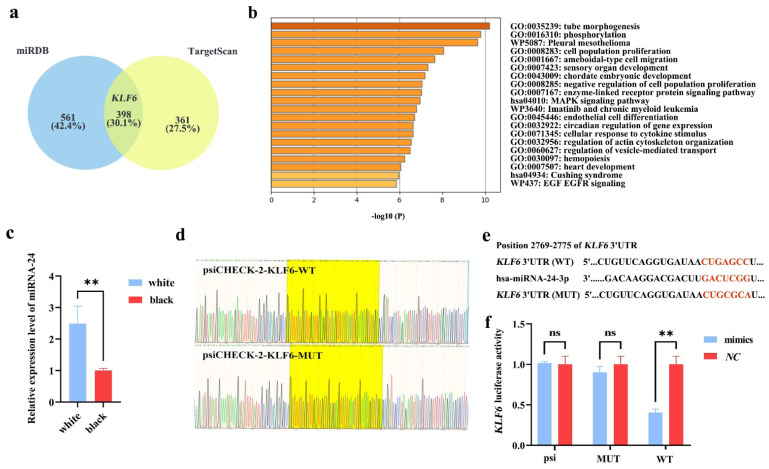
Validation of MiRNA-24 cashmere color correlation and targeting relationship. (a) Target gene screening based on miRDB and Target Scan databases. (b) Target gene GO/KEGG enrichment analysis chart. (c) The expression level of miRNA-24 in the skin of black and white Cashmere goats (** p<0.01). (d) The bacterial liquid sequencing image of the reconstructed plasmid shows the binding sequences of psiCHECK-2-KLF6-WT and psiCHECK-2-KLF6-MUT 3'UTR, marked in yellow. (e) The point mutation design of KLF6-WT and KLF6-MUT 3'UTR binding sequences, with binding sites marked in red. (f) The result of dual luciferase assay (** p<0.01). WT, wild-type; MUT, mutant-type.

The relationship between miRNA-24 and the coat color of the Cashmere goat was confirmed by RT-qPCR. The findings demonstrated that, in Cashmere goat skin tissues of black and white, which had significant differences in melanin concentration, miRNA-24 was highly expressed in the white skin tissues relative to the black skin tissues (** p<0.01) (Figure 1A), suggesting the connection between miRNA-24 and coat color of Cashmere goat. TargetScan, an online bioinformatics website, and miRDB were used to initially determine the interacting genes of miRNA-24-3p. As seen in Figure 1B, the results indicated that 398 genes, including *KLF6*, were co-targeted by miRNA-24 in two databases. As seen in Figure 1C, GO/KEGG enrichment analysis revealed that the target gene is implicated in the MAPK signaling pathway (hsa04010), phosphorylation (GO: 0016310), and biological processes related to cell growth and proliferation. Among these, *KLF6* has been linked to the melanin synthesis process, which facilitates melanogenesis [23]. Consequently, *KLF6* was finally selected as miRNA-24’s target gene.

### Confirmation of interaction between miRNA-24 and *KLF6*

The effective construction of the reconstructed vector was shown by the precise display of both wild-type and mutant sequences of the target gene in the bacterial liquid sequencing map (Figure 1D). The dual luciferase detection system’s findings demonstrated that miRNA-24 significantly affected *KLF6* expression. The binding sequence sites design of *KLF6* 3’UTR wild-type and mutant-type was shown in Figure 1E. When psiCHECK-2-KLF6-WT was co-transfected with miRNA-24 mimics, the relative activity of luciferase was significantly lower than with miRNA-24 *NC*. As seen in Figure 1F, however, there were no significant differences in the relative activity of firefly luciferase when miRNA-24 mimics and miRNA-24 *NC* were co-transfected with psiCHECK-2-KLF6-MUT and psiCHECK-2 empty plasmids (** p<0.01). Following the findings, miRNA-24 targets *KLF6*.

### The regulation of *KLF6* expression by miRNA-24 during melanogenesis

**Figure 2:**
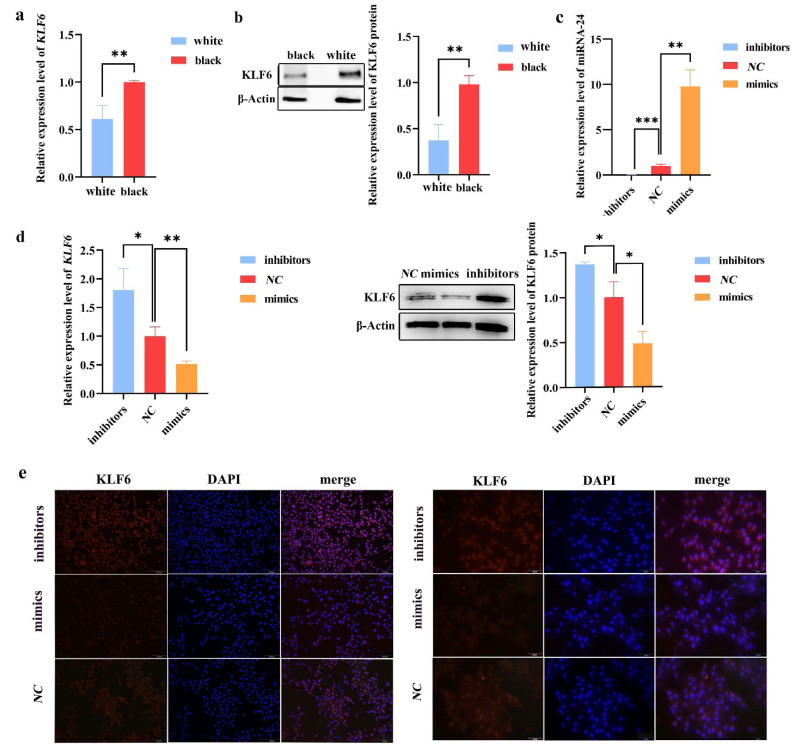
MiRNA-24 regulates the expression of *KLF6*. (a) *KLF6* is relatively expressed in the skin of black and white Cashmere goats (** p<0.01). (b) Relative protein expression and bands of *KLF6* in black and white Cashmere goat skin (N = 3, ** p<0.01). (c) The relative gene expression of miRNA-24 in B16-F10 cells transfected with miRNA-24 mimics/inhibitors/NC. (d) Relative gene and protein expression of *KLF6* in B16-F10 cells transfected with miRNA-24 mimics/inhibitors/*NC* (N = 3, * p<0.05, ** p<0.01). (e) The protein localization and expression of KLF6 in B16-F10 cells in each group, as shown in the left figure ×40 and the right figure ×1,000. N, number of biological repeated experiments.

The results of the RT-qPCR experiment showed that *KLF6* was expressed differently in the skin of black and white Cashmere goats (** p<0.01) (Figure 2A), suggesting a potential connection between *KLF6* and the formation of coat color. Additionally, the expression of *KLF6* in black skin tissue was considerably higher than that in white skin tissue, which went against the trend of miRNA-24 expression in Cashmere goat skin with different cashmere colors. The outcomes of the Western Blotting experiment further demonstrated that the white skin tissue had significantly lower *KLF6* protein expression than the black skin tissue (** p<0.01). Figure 2B, Supplement 1 displays the protein bands and visualization data. According to the above research, miRNA-24 may negatively regulate *KLF6*. Further in vitro investigations were carried out to confirm this finding. As seen in Figure 2C (** p<0.01, *** p<0.001), transfection of miRNA-24 mimics and inhibitors into B16-F10 cells significantly increased and inhibited miRNA-24 expression compared to transfection of miRNA-24 *NC*. This result indicated a good transfection effect. Furthermore, as Figure 2D, Supplement 2 (* p<0.05, ** p<0.01) illustrated, the expression of the *KLF6* was considerably higher with transfection of miRNA-24 inhibitors than with miRNA-24 mimics. It demonstrated that miRNA-24 had a negative regulatory effect on *KLF6* expression during melanogenesis. Immunofluorescence experiments showed the expression and localization of KLF6 in B16-F10 under ×1,000 and ×40 fluorescence microscopes. The red fluorescence labeled target KLF6 protein was mainly localized in B16-F10. The blue fluorescence labeled the cell nucleus. Compared with the miRNA-24 *NC* group, the KLF6 protein in the miRNA-24 mimics showed weaker fluorescence signals, but the KLF6 protein in the miRNA-24 inhibitors group showed stronger fluorescence signals, further verifying the negative regulatory effect of miRNA-24 on KLF6, as shown in Figure 2E.

### *KLF6* mediates STAT3 protein and phosphorylation expression, affecting *TYR* expression

**Figure 3:**
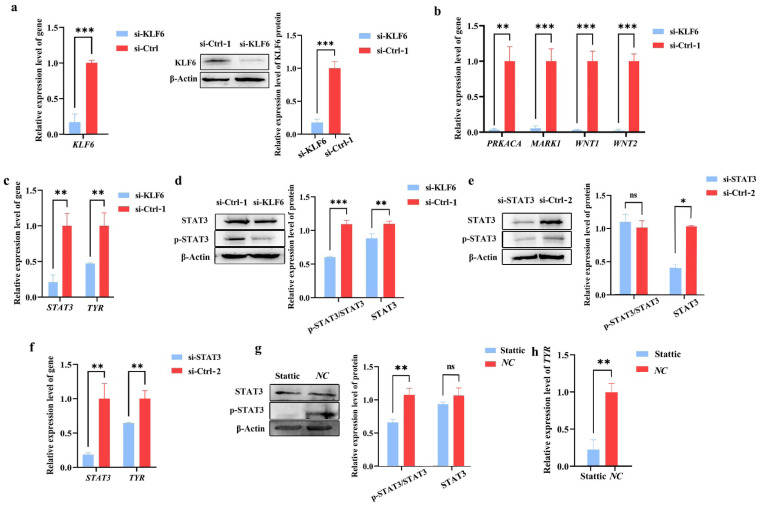
The mechanism by which *KLF6* affects melanogenesis. (a) After silencing *KLF6* (si-KLF6), the relative expression levels of *KLF6* gene and protein (N = 3, *** p<0.001). (b) The mRNA levels of genes related to the melanogenesis pathway (** p<0.01, *** p<0.001). (c) The mRNA levels of *STAT3* and *TYR* after silencing *KLF6* (** p<0.01). (d) After *KLF6* silencing, STAT3 protein and its phosphorylation levels (N = 3, ** p<0.01, *** p<0.001). (e) After *STAT3* silencing, the expression levels of STAT3 protein and its phosphorylation (N = 3, * p<0.05). (f) The expression levels of *STAT3* and *TYR* mRNA after *STAT3* silencing (** p<0.01). (g) The protein and phosphorylation levels after STAT3 phosphorylation inhibition (N = 3, ** p<0.01). (h) The mRNA expression level of *TYR* after STAT3 phosphorylation inhibition (** p<0.01). N, number of biological repeated experiments.

After silencing *KLF6* expression in B16-F10 cells, compared with the si-Ctrl-1 group, the *KLF6* gene and protein levels in the si-KLF6 group were significantly reduced (*** p<0.001), as shown in Figure 3A, Supplement 3, indicating a good silencing effect. The RT-qPCR experiment results showed that compared with the si-Ctrl-1 group, the expression of *PRKACA, MAPK1, WNT1*, and *WNT2* genes related to the melanin generation pathway in the si-KLF6 group was significantly reduced (** p<0.01, *** p<0.001), as shown in Figure 3B. The results indicated that *KLF6* affects melanin generation by affecting related genes in the melanin generation pathway.

After silencing *KLF6* expression in B16-F10 cells, the expression levels of *STAT3* and *TYR* genes were significantly reduced in the si-KLF6 group compared to the si-Ctrl-1 group, as shown in Figure 3C (** p<0.01). At the same time, compared with the si-Ctrl-1 group, the total protein level of STAT3 in the si-KLF6 group was significantly reduced, and its phosphorylation modification was further inhibited, resulting in a significant decrease in the p-STAT3/STAT3 ratio, as shown in Figure 3D, Supplement 3 (** p<0.01, *** p<0.001), indicating that *KLF6* may promote STAT3 protein expression, increase its phosphorylation level and affect Melanogenesis. Compared with the si-Ctrl-2 group, there was no significant difference in STAT3 phosphorylation levels in the si-STAT3 group. Both mRNA and total protein levels were significantly reduced (* p<0.05, ** p<0.01), and *TYR* gene levels were subsequently reduced (** p<0.01), as shown in Figures 3E, 3F, Supplement 4. After administering Stattic (1.25 μM, 0.1% DMSO) to B16-F10 cells for 24 h, there was no significant difference in STAT3 total protein levels compared to the *NC* group (0.1% DMSO), and its phosphorylation level was significantly reduced. *TYR* expression levels were also significantly reduced, as shown in Figures 3G, 3H, Supplement 5 (** p<0.01), indicating that STAT3 protein expression and phosphorylation affect the final melanogenesis. In summary, the research results indicated that *KLF6* affects the expression of the *TYR* and ultimately affects melanogenesis by mediating STAT3 protein expression and increasing its phosphorylation level.

### MiRNA-24 regulates melanogenesis in BALB/c skin by downregulating *KLF6*

**Figure 4:**
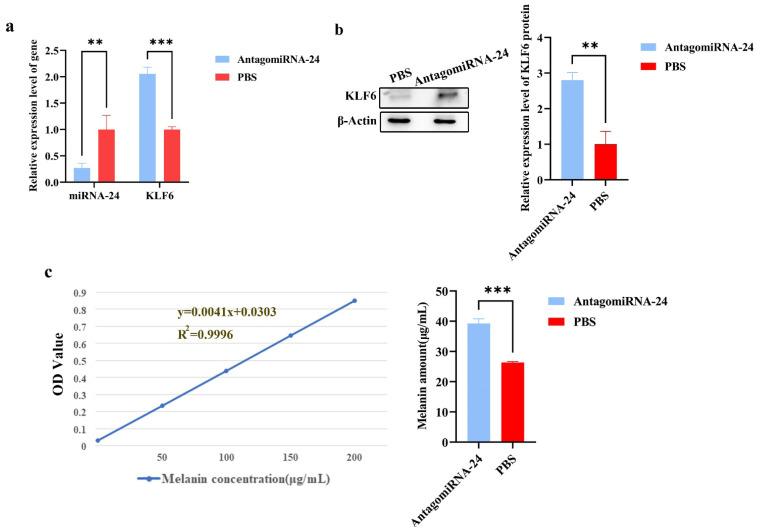
The animal replenishment experiment. (a) The relative gene expressions of miRNA-24 and *KLF6* in the AntagomiRNA-24 and PBS (control) group (** p<0.01, *** p<0.001). (b) The relative protein levels of *KLF6* in the AntagomiRNA-24 and PBS group (N = 3, ** p<0.01). (c) The standard curve of melanin on the left and the melanin contents in the skin of the AntagomiRNA-24 and PBS group (*** p<0.001). PBS, phosphate-buffered saline; OD, optical density; N, number of biological repeated experiments.

As demonstrated in Figure 4A, the experiment revealed that the expression of miRNA-24 was significantly inhibited in the AntagomiRNA-24 group (** p<0.01) as compared to the PBS control group based on the RT-qPCR results. However, *KLF6* had an opposing trend to miRNA-24 expression (*** p<0.001). The *KLF6* protein expression was increased in the AntagomiRNA-24 group (** p<0.01) (Figure 4B, Supplement 6), which is in accordance with the results of the RT-qPCR. The experiment outcomes demonstrated that miRNA-24 downregulated *KLF6* expression during melanogenesis. The melanin content was measured and demonstrated a significant increase of melanin content in the AntagomiRNA-24 group compared to the PBS group (*** p<0.001) (Figure 4C). This suggested that miRNA-24 inhibited *KLF6* to affect melanogenesis.

## DISCUSSION

A great breed in China that produces both cashmere and meat is the Liaoning Cashmere Goat. It has significant practical and financial significance and possesses the qualities of long cashmere fiber, fine cashmere, high cashmere output, and stable genetics. Apart from the superior qualities of cashmere fibers, such as their strength and length, the variety of colors are also gaining more and more attention. In addition to guaranteeing the quality of cashmere items, using natural cashmere as a raw material also helps to prevent the negative health effects of dyed fur. However, the disadvantages of Cashmere goats, such as their genetic instability and single natural cashmere color, limit the expansion of the cashmere industry. Therefore, it is valuable to artificially regulate cashmere color. Gene regulation is one method of artificially controlling cashmere color. In this study, animal skin and cashmere color generation mechanisms were extensively studied using miRNA, a crucial molecular tool for controlling melanogenesis. This study not only elucidated the mechanism by which miRNA-24 downregulates *KLF6* and affects melanogenesis, and provided theoretical support for the color tone control of the coat color of the Cashmere goat.

According to relevant research reports, miRNA-24 can participate in various regulatory processes of cell growth and development, and its mechanism of action is achieved by acting on signaling pathways such as Wnt/β-catenin and MAPK. However, its mechanism of action on melanocyte maturation and pigment production is still unclear. In this study, following expression suppression of miRNA-24 by antagomiRNA-24, a crucial component in the transcriptional translation control of cellular genes, increased the amount of melanin in BALB/c mice, suggesting a relationship between miRNA-24 and melanogenesis. As a transcription activator of genes, *KLF6* belongs to the Kruppel-like transcription factor family. In addition to splicing, it is generally thought to have anti-cancer properties. Apart from its correlation with cancer development, *KLF6* has been demonstrated to have a role in several processes, including inflammation, cell proliferation and apoptosis, and body growth and development. Macrophage inflammation and hypoxia response are caused by *KLF6*’s promotion of HIF-1α expression, which increases glycolysis, cellular hypoxia, and inflammatory gene expression [24]. Furthermore, mesenchymal stem cells’ extracellular vesicle component, miRNA-148a-5p, alters the polarization status of macrophages, decreases the number of inflammatory cells, and ameliorates liver fibrosis via downregulating *KLF6* expression and influencing *STAT3* phosphorylation in the JAK/STAT pathway [25]. According to recent research, *KLF6* is linked to melanosome maturation. CRISPR-Cas9 knockout mice show a marked reduction in melanin in their hair following *in vivo* deletion of the *KLF6* gene, leading to phenotypes like hair whitening. Studies have shown that gene deletion does not affect the number of melanocytes but the final melanin content by affecting the number of mature melanosomes [23]. This suggests a close relationship between *KLF6* and melanogenesis, although the exact mechanism of action is yet unknown.

This study explored the regulatory effect of miRNA-24 on *KLF6* from both *in vivo* and *in vitro* perspectives, using B16-F10 cells instead of melanocytes *in vitro*. Commonly employed in melanin synthesis model studies, B16-F10 cells are a clone subtype of mouse skin B16 melanoma cells that show strong melanogenesis levels in high glucose DMEM medium. In 2024, Yang et al used pigmentation enhancers to develop a model of improved melanin synthesis based on B16-F10 cells [26]. In 2019, Lee et al examined the effects of luteolin 7-sulfate on *MITF* and *CREB* levels in Melanogenesis using B16-F10 cells [27]. This study examined the targeted connection between miRNA-24 and *KLF6 in vitro* using B16-F10 cells as a stand-in for melanocytes. Melanocytes can be used for further confirmation.

To further investigate the melanin pathway-related genes involved in the action of *KLF6*. By transfecting siRNA, *KLF6* expression was suppressed, the mRNA level of TYR was reduced, and the STAT3 protein and its phosphorylation were decreased. It has been demonstrated that the transcription activator STAT3 protein controls tyrosinase activity via unconventional mechanisms [12]. Our study discovered that suppressing STAT3 protein and phosphorylation expression both decreased *TYR* levels. Thus, *KLF6* influences the final *TYR* level in melanosomes through the downregulation of STAT3 expression, which impacts melanogenesis and compensates for the mechanistic study of *KLF6*’s function in melanin formation. Simultaneously, the downregulation of genes implicated in the traditional route of melanogenesis, including *WNT1, WNT2, PRKACA*, and *MAPK1*, results from *KLF6* silencing. This finding implied that *KLF6* could affect the melanin synthesis process via additional pathways. For example, the MC1R/α-MSH signaling pathway involving the *PRKACA*, the Ras-Raf-MEK-ERK cascade activation mediated by *MAPK1*, and the classic Melanogenesis regulatory pathway of Wnt/β-catenin involving *WNT1* and *WNT2*. It should be noted that previous studies have reported that silencing *KLF6* leads to a decrease in STAT3 phosphorylation levels in macrophages, but does not alter their total protein expression [12]. In this study, silencing *KLF6* not only affects STAT3 phosphorylation levels but also significantly reduces STAT3 total protein levels, indicating that differences in cell types may result in different regulatory mechanisms. This also reveals that *KLF6* affects *TYR* levels through two independent pathways: promoting STAT3 protein expression and increasing its phosphorylation levels.

## CONCLUSION

In summary, miRNA-24 has been confirmed to be associated with the color formation of cashmere wool. It affects the expression of genes related to the melanin synthesis pathway (*PRKACA, MAPK1, WNT1, WNT2, STAT3*) by downregulating *KLF6* expression, thereby mediating the expression and phosphorylation level of STAT3 protein to regulate the process and final content of melanin synthesis in animal fur.

## Figures and Tables

**Table 1:** Primer sequences of real-time PCR for miRNA-24

Genes	Primer	Primer sequence (5′–3′)
*miRNA-24-3p*	RT-primer	GTCGTATCCAGTGCAGGGTCCGAGGTATTCGCACTGGATACGACGTTCCT
	F-primer	CGCGTGGCTCAGTTCAGC
	R-primer	AGTGCAGGGTCCGAGGTATT
*U6*	RT-primer	CGCTTCACGAATTTGCGTGTCAT
	F-primer	GCTTCGGCAGCACATATACTAAAAT
	R-primer	CGCTTCACGAATTTGCGTGTCAT

PCR, polymerase chain reaction; RT, stem-loop RT primer; F, forward primer; R, reverse primer.

**Table 2:** Primer sequences of real-time PCR for genes

Genes	Primer	Primer sequence (5′–3′)
*KLF6*	F-primer	GCACGCCTCCCTGTTTTTAC
(*Capra hircus*)	R-primer	TACAACTGCCGAGACACCAG
*KLF6*	F-primer	GTGCATGGTGTTTGGGTGAC
(*Mus musculus*)	R-primer	TCAACACAACCATCCCACCC
*STAT3*	F-Primer	CATCCTGAAGCTGACCCAGG
(*Mus musculus*)	R-Primer	TCCTCACATGGGGGAGGTAG
*TYR*	F-Primer	CGGCCAACGATCCCATTTTTC
(*Mus musculus*)	R-Primer	GACTTTGAGCTGAATTGCCAGG
*MAPK1*	F-Primer	ATTACGACCCGAGTGACGAG
(*Mus musculus*)	R-Primer	AGGACCAGGGGTCAAGAACT
*PRKACA*	F-Primer	TTTCAAGCCTGTTTCCTGGGT
(*Mus musculus*)	R-Primer	ATGTTGAAACTTCCCCGGCA
*WNT1*	F-Primer	ACTGCACGAGTGTCTGTGAG
(*Mus musculus*)	R-Primer	TGCTAGCGAGTCTGTTTGGG
*WNT2*	F-Primer	TGGAATTGCAACACCCTGGA
(*Mus musculus*)	R-Primer	TTGGCGCTTCCCATCTTCTT
*GAPDH*	F-Primer	CCGTAACTTCTGTGCTGTGCC
(*Capra hircus*)	R-Primer	TGAAGGGGTCATTGATGGCAAC
*GAPDH*	F-primer	AAGAGGGATGCTGCCCTTAC
(*Mus musculus*)	R-primer	GTTCACACCGACCTTCACCA

PCR, polymerase chain reaction; F, forward primer; R, reverse primer.

**Table 3:** Primers for gene annealing connection

Gene	Primer	Primer sequence (5′–3′)
*KLF6*	WT-F-Primer	TCGAGCTGTTCAGGTGATAACTGAGCCTCAATCAAGCAGAAAGC
	WT-R-Primer	GGCCGCTTTCTGCTTGATTGAGGCTCAGTTATCACCTGAACAGC
	MUT-F-Primer	TCGAGCTGTTCAGGTGATAACTGCGCATCAATCAAGCAGAAAGC
	MUT-R-Primer	GGCCGCTTTCTGCTTGATTGATGCGCAGTTATCACCTGAACAGC

WT, wild-type; F, forward primer; R, reverse primer; MUT, mutation-type.

## SUPPLEMENTARY MATERIAL

**Supplement 1**. The parallelism verification of KLF6 protein expression in black and white skin of Cashmere goats.

**Supplement 2**. The parallelism validation of KLF6 protein expression in B16-F10 cells transfected with miRNA-24 mimics, inhibitors, and NC.

**Supplement 3**. The parallel validation of protein expression of KLF6, STAT3, and phosphorylated STAT3 proteins after silencing KLF6.

**Supplement 4**. The parallel validation of STAT3 protein expression.

**Supplement 5**. Parallel validation of protein expression after STAT3 protein phosphorylation inhibition.

**Supplement 6**. The parallel validation of KLF6 protein expression after subcutaneous injection of antagomiRNA-24 into BALB/C mice.

## References

[1] Sun Q, Lee W, Hu H (2023). Dedifferentiation maintains melanocyte stem cells in a dynamic niche. Nature.

[2] Pavan WJ, Sturm RA (2019). The genetics of human skin and hair pigmentation. Annu Rev Genomics Hum Genet.

[3] Ohbayashi N, Fukuda M (2020). Recent advances in understanding the molecular basis of melanogenesis in melanocytes. F1000Research.

[4] Weiner L, Fu W, Chirico WJ, Brissette JL (2014). Skin as a living coloring book: how epithelial cells create patterns of pigmentation. Pigment Cell Melanoma Res.

[5] Dumbuya H, Hafez SY, Oancea E (2020). Cross talk between calcium and ROS regulate the UVA-induced melanin response in human melanocytes. FASEB J.

[6] Adelmann CH, Traunbauer AK, Chen B (2020). MFSD12 mediates the import of cysteine into melanosomes and lysosomes. Nature.

[7] Kim J, Hong SC, Lee EH, Lee JW, Yang SH, Kim JC (2021). Preventive effect of M. Cochinchinensis on melanogenesis via tyrosinase activity inhibition and p-PKC signaling in melan-A cell. Nutrients.

[8] O'Carroll D, Schaefer A (2013). General principals of miRNA biogenesis and regulation in the brain. Neuropsychopharmacology.

[9] Fabian MR, Sonenberg N, Filipowicz W (2010). Regulation of mRNA translation and stability by microRNAs. Annu Rev Biochem.

[10] Chen S, Nie H, Huo Z, Yan X (2024). Comprehensive analysis of differentially expressed mRNA, lncRNA and miRNA, and their ceRNA networks in the regulation of shell color in the Manila clam (Ruditapes philippinarum). Int J Biol Macromol.

[11] Bertrand JU, Petit V, Aktary Z (2024). Loss of dicer in newborn melanocytes leads to premature hair graying and changes in integrin expression. J Invest Dermatol.

[12] Shin SY, Gil HN, Choi JH, Lim Y, Lee YH (2018). Agerarin inhibits α-MSH–induced TYR gene transcription via STAT3 suppression independent of CREB-MITF pathway. J Dermatol Sci.

[13] Wang XY, Guan XH, Yu ZP (2021). Human amniotic stem cells-derived exosmal miR-181a-5p and miR-199a inhibit melanogenesis and promote melanosome degradation in skin hyperpigmentation, respectively. Stem Cell Res Ther.

[14] Li J, Ba X, Li J (2022). MicroRNA-200a regulates skin pigmentation by targeting WNT5A and FZD4 in cashmere goats. Res Vet Sci.

[15] Yang G, Wu M, Liu X (2022). MiR-24-3p conservatively regulates muscle cell proliferation and apoptosis by targeting common gene CAMK2B in rat and cattle. Animals.

[16] Wang Q, Huang Z, Xue H (2008). MicroRNA miR-24 inhibits erythropoiesis by targeting activin type I receptor ALK4. Blood.

[17] Liu F, Zhang X, Peng Y (2021). miR-24 controls the regenerative competence of hair follicle progenitors by targeting Plk3. Cell Rep.

[18] Amelio I, Lena AM, Bonanno E, Melino G, Candi E (2013). miR-24 affects hair follicle morphogenesis targeting Tcf-3. Cell Death Dis.

[19] Bian Q, Chen B, Weng B (2021). circBTBD7 promotes immature porcine sertoli cell growth through modulating miR-24-3p/MAPK7 axis to inactivate p38 MAPK signaling pathway. Int J Mol Sci.

[20] Lopez JP, Fiori LM, Cruceanu C (2017). MicroRNAs 146a/b-5 and 425-3p and 24-3p are markers of antidepressant response and regulate MAPK/Wnt-system genes. Nat Commun.

[21] Jin F, Yang R, Wei Y (2019). HIF-1α-induced miR-23a~27a~24 cluster promotes colorectal cancer progression via reprogramming metabolism. Cancer Lett.

[22] Otmani K, Rouas R, Lagneaux L (2023). Acute myeloid leukemia-derived exosomes deliver miR-24-3p to hinder the T-cell immune response through DENN/MADD targeting in the NF-κB signaling pathways. Cell Commun Signal.

[23] Bajpai VK, Swigut T, Mohammed J (2023). A genome-wide genetic screen uncovers determinants of human pigmentation. Science.

[24] Kim GD, Ng HP, Chan ER, Mahabaleshwar GH (2020). Kruppel-like factor 6 promotes macrophage inflammatory and hypoxia response. FASEB J.

[25] Tian S, Zhou X, Zhang M (2022). Mesenchymal stem cell-derived exosomes protect against liver fibrosis via delivering miR-148a to target KLF6/STAT3 pathway in macrophages. Stem Cell Res Ther.

[26] Yang Y, Lv Z, An Q (2024). Tricholoma matsutake polysaccharides suppress excessive melanogenesis via JNK-mediated pathway: investigation in 8-methoxypsoralen induced B16–F10 melanoma cells and clinical study. Heliyon.

[27] Lee SW, Kim JH, Song H, Seok JK, Hong SS, Boo YC (2019). Luteolin 7-sulfate attenuates melanin synthesis through inhibition of CREB- and MITF-mediated tyrosinase expression. Antioxidants.

