# Supplementary material for: MiRNA-24 downregulates KLF6 affecting STAT3 protein expression and phosphorylation regulating melanogenesis in cashmere goat coat
Source: Anim Biosci. 2025 Jun 10;38(9):1984–95. doi: 10.5713/ab.24.0824 (PMC12415448; doi:10.5713/ab.24.0824)

Supplement 1. The parallelism verification of KLF6 protein expression in black and white skin of Cashmere goats. a. The KLF6 Polyclonal Antibody (Proteintech) product predicts 32-42 kDa protein sizes. b. KLF6 levels in the Cashmere goat skin of white and black.

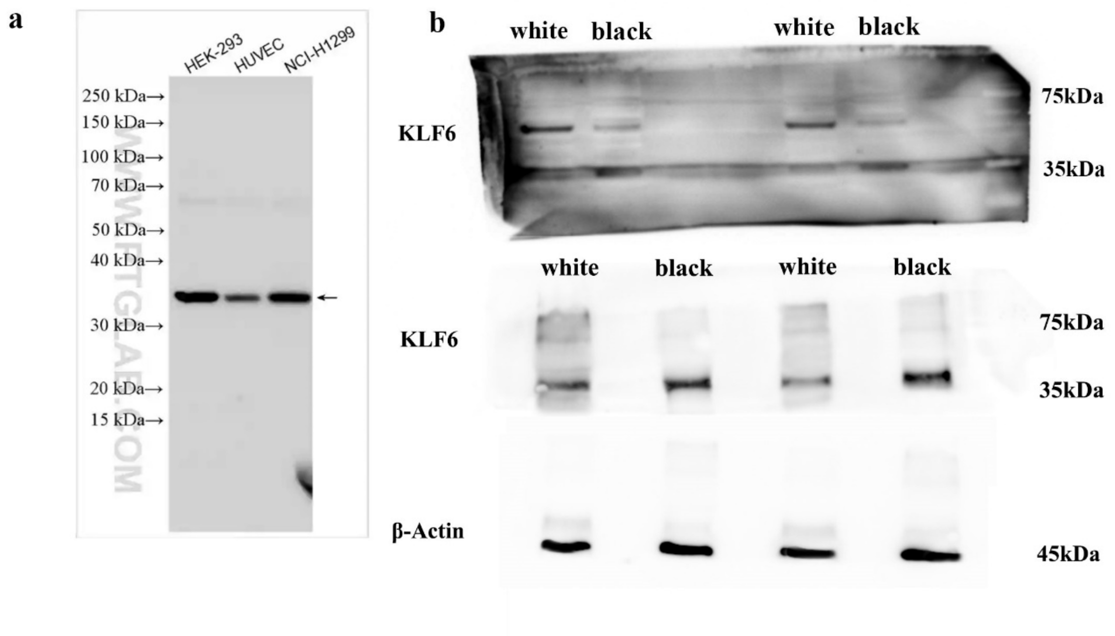

Supplement: Supplementary file 1 [file ab-24-0824-Supplementary-1.pdf]
