# Supplementary material for: MiRNA-24 downregulates KLF6 affecting STAT3 protein expression and phosphorylation regulating melanogenesis in cashmere goat coat
Source: Anim Biosci. 2025 Jun 10;38(9):1984–95. doi: 10.5713/ab.24.0824 (PMC12415448; doi:10.5713/ab.24.0824)

Supplement 2. The parallelism validation of KLF6 protein expression in B16-F10 cells transfected with miRNA-24 mimics, inhibitors, and NC. a. The KLF6 Monoclonal Antibody (Proteintech) product predicts a protein size of 32-42 kDa. b. KLF6 levels in the mimics, inhibitors, and NC groups.

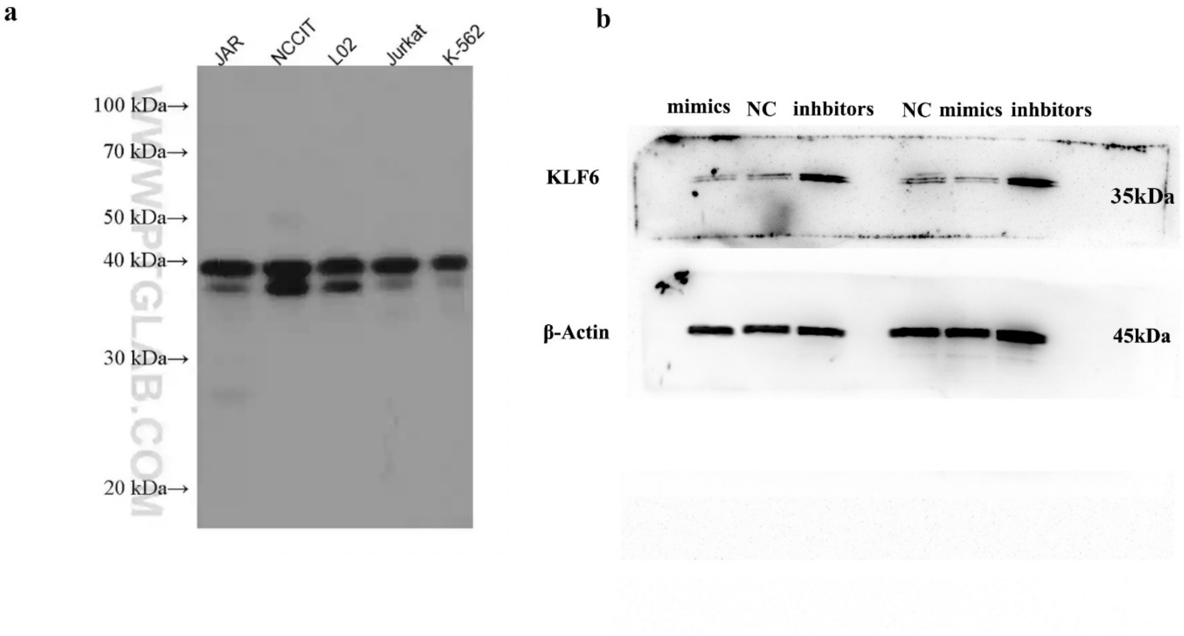

Supplement: Supplementary file 2 [file ab-24-0824-Supplementary-2.pdf]
