# Supplementary material for: MiRNA-24 downregulates KLF6 affecting STAT3 protein expression and phosphorylation regulating melanogenesis in cashmere goat coat
Source: Anim Biosci. 2025 Jun 10;38(9):1984–95. doi: 10.5713/ab.24.0824 (PMC12415448; doi:10.5713/ab.24.0824)

Supplement 3. The parallel validation of protein expression of KLF6, STAT3, and phosphorylated STAT3 proteins after silencing KLF6. a. The predicted protein size of STAT3 antibody (Abmart) and STAT3 (phosphor Ser727) antibody (Abmart) products is 88kDa. b. KLF6, STAT3, and P-STAT3 levels in the si-KLF6 and si-Ctrl groups. Red clipping box: p-STAT3 band approximately 88kDa in size.

a

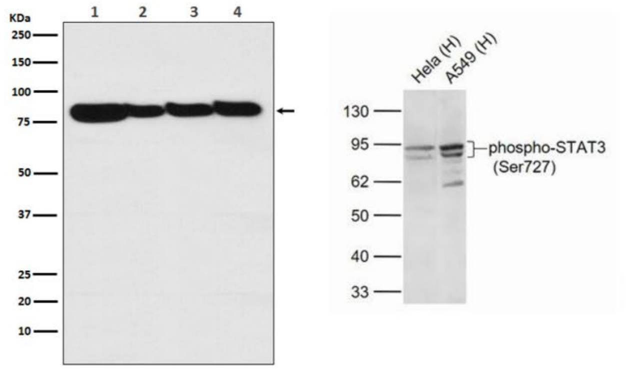

b

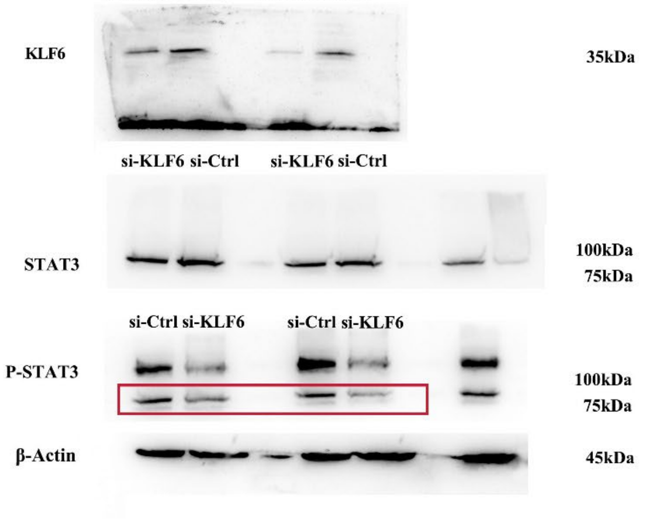

Supplement: Supplementary file 3 [file ab-24-0824-Supplementary-3.pdf]
