# Supplementary material for: MiRNA-24 downregulates KLF6 affecting STAT3 protein expression and phosphorylation regulating melanogenesis in cashmere goat coat
Source: Anim Biosci. 2025 Jun 10;38(9):1984–95. doi: 10.5713/ab.24.0824 (PMC12415448; doi:10.5713/ab.24.0824)

Supplement 4. The parallel validation of STAT3 protein expression. a. The predicted protein size of STAT3 antibody (Abmart) and STAT3 (phosphor Ser727) antibody (Abmart) products is 88kDa. b. P-STAT3 and STAT3 levels were in the si-Ctrl and the si-STAT3 group. Red clipping box: p-STAT3 band approximately 88kDa in size.

a

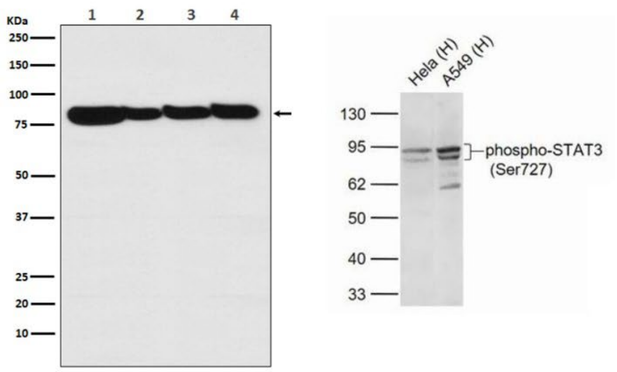

b

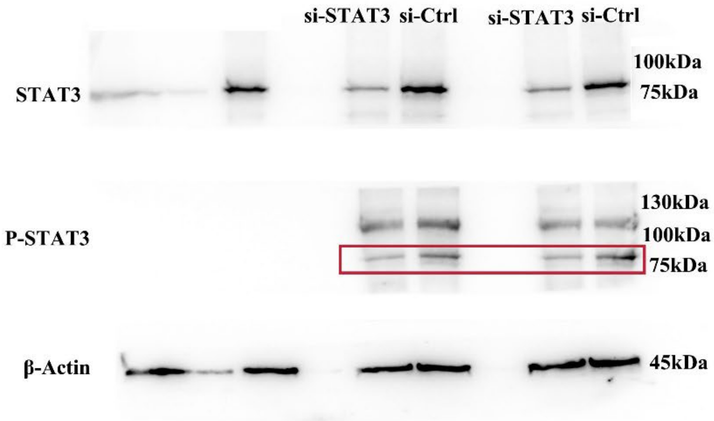

Supplement: Supplementary file 4 [file ab-24-0824-Supplementary-4.pdf]
