# Supplementary material for: MiRNA-24 downregulates KLF6 affecting STAT3 protein expression and phosphorylation regulating melanogenesis in cashmere goat coat
Source: Anim Biosci. 2025 Jun 10;38(9):1984–95. doi: 10.5713/ab.24.0824 (PMC12415448; doi:10.5713/ab.24.0824)

Supplement 5. Parallel validation of protein expression after STAT3 protein phosphorylation inhibition. a. The predicted protein size of STAT3 antibody (Abmart) and STAT3 (phosphor Ser727) antibody (Abmart) products is 88kDa. b. P-STAT3 and STAT3 levels were in the DMSO group (NC) and the Stattic group. Red clipping box: p-STAT3 band approximately 88kDa in size.

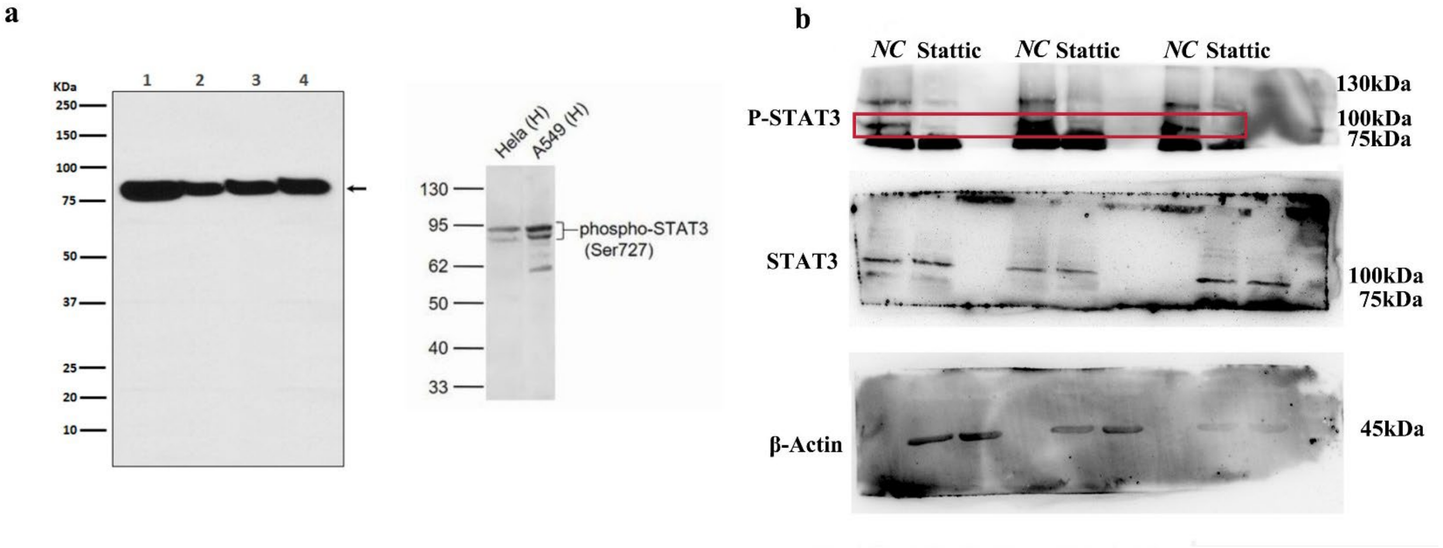

Supplement: Supplementary file 5 [file ab-24-0824-Supplementary-5.pdf]
