# Supplementary material for: MiRNA-24 downregulates KLF6 affecting STAT3 protein expression and phosphorylation regulating melanogenesis in cashmere goat coat
Source: Anim Biosci. 2025 Jun 10;38(9):1984–95. doi: 10.5713/ab.24.0824 (PMC12415448; doi:10.5713/ab.24.0824)

Supplement 6. The parallel validation of KLF6 protein expression after subcutaneous injection of antagomiRNA-24 into BALB/C mice. a. The KLF6 Polyclonal Antibody (Proteintech) product predicts 32-42 kDa protein sizes. b. The expression of KLF6 protein in the AntagomiRNA-24 and PBS groups.

a

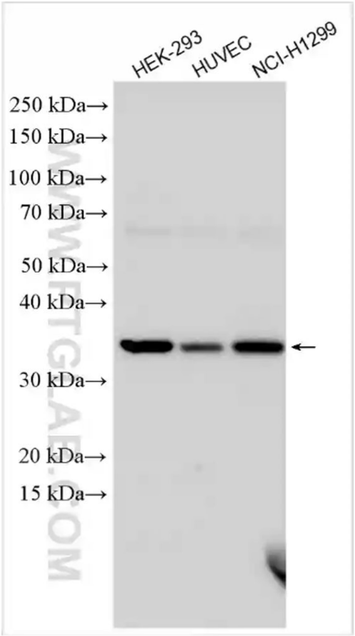

b

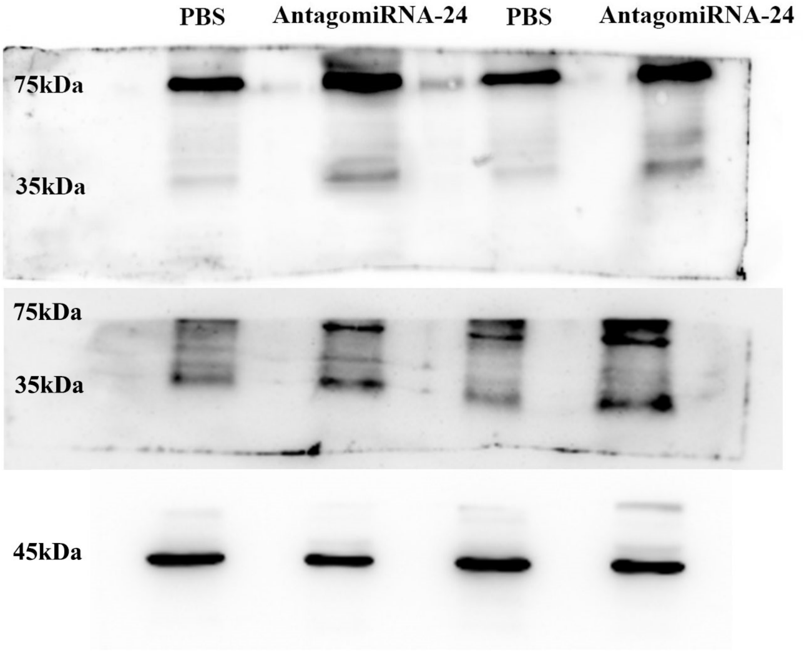

Supplement: Supplementary file 6 [file ab-24-0824-Supplementary-6.pdf]
